# Estimation of elbow flexion torque using equilibrium optimizer on feature selection of NMES MMG signals and hyperparameter tuning of random forest regression

**DOI:** 10.3389/fresc.2025.1469797

**Published:** 2025-02-19

**Authors:** Raphael Uwamahoro, Kenneth Sundaraj, Farah Shahnaz Feroz

**Affiliations:** ^1^ Regional Centre of Excellence in Biomedical Engineering and E-Health, University of Rwanda, Kigali, Rwanda; ^2^ Fakulti Teknologi dan Kejuruteraan Elektronik dan Komputer, Universiti Teknikal Malaysia Melaka, Melaka, Malaysia

**Keywords:** neuromuscular electrical stimulation, mechanomyography, joint torque estimation, machine learning, general learning equilibrium optimizer

## Abstract

**Background:**

The assessment of limb joint torque is essential for understanding musculoskeletal system dynamics. Yet, the lack of direct muscle strength measurement techniques has prompted previous research to deploy joint torque estimation using machine learning models. These models often suffer from reduced estimation accuracies due to the presence of redundant and irrelevant information within the rapidly expanding complex biomedical datasets as well as suboptimal hyperparameters configurations.

**Methods:**

This study utilized a random forest regression (RFR) model to estimate elbow flexion torque using mechanomyography (MMG) signals recorded during electrical stimulation of the biceps brachii (BB) muscle in 36 right-handed healthy subjects. Given the significance of both feature engineering and hyperparameter tuning in optimizing RFR performance, this study proposes a hybrid method leveraging the General Learning Equilibrium Optimizer (GLEO) to identify most informative MMG features and tune RFR hyperparameters. The performance of the GLEO-coupled with the RFR model was compared with the standard Equilibrium Optimizer (EO) and other state-of-the-art algorithms in physical and physiological function estimation using biological signals.

**Results:**

Experimental results showed that selected features and tuned hyperparameters demonstrated a significant improvement in root mean square error (RMSE), coefficient of determination (R^2^) and slope with values improving from 0.1330 to 0.1174, 0.7228 to 0.7853 and 0.6946 to 0.7414, respectively for the test dataset. Convergence analysis further revealed that the GLEO algorithm exhibited a superior learning capability compared to EO.

**Conclusion:**

This study underscores the potential of the hybrid GLEO approach in selecting highly informative features and optimizing hyperparameters for machine learning models. These advancements are essential for evaluating muscle function and represent a significant advancement in musculoskeletal biomechanics research.

## 1 Introduction

Joint torque is a critical measure of skeletal muscle function (1). This parameter reflects the degree of muscle activation, which holds substantial importance in clinical studies of muscle strength (2). For instance, studies have shown that during muscle education, improved muscle strength is closely associated with functional outcomes (3). However, assessment of muscle performance under low-efforts conditions remains largely underexplored.

Previous studies have primarily relied on manual muscle testing to examine muscle capacity. Yet, its reliability heavily depends on the tester's proficiency, making it unsuitable for low muscle activity (4). At the joint level, isokinetic dynamometers can quantify the mechanical loads by measuring the joint torque produced by synergistic muscle groups. Nevertheless, their applicability is limited to fixed laboratory setups, underscoring the need for alternative methods capable of real-time quantification of joint torque measurement and bioelectric potentials generated during muscle contraction.

One of neuromuscular function screening is surface electromyography (sEMG) (4), which quantifies the electrical activity of the muscle used for joint torque estimation. However, previous studies have predominantly focused on high efforts of muscle contraction experiments, which render the interpretation of sEMG not generalizable at low muscle activation accounted for in degenerative conditions of muscles caused by ageing, trauma, or neuromuscular disorders (5). In addition, sEMG is prone to artefacts from recording equipment, body motion, and tremors. These results compromise the effectiveness of sEMG for real time physical activity estimation in upper limb exoskeleton control (6).

In response to these limitations, recent research has explored mechanomyography (MMG) as a non-invasive technique that records the lateral oscillations of muscle fibers providing a mechanical counterpart to electromyography (EMG) generated by the contraction of skeletal muscle fibers (7). MMG reflects both the magnitude and patterns of motor unit recruitment, as well as the rate coding accounted for during the force-production typically measured by the root mean square (RMS) value and mean power frequency (MPF). Specifically, analysis of the RMS and MPF from the biceps brachii (BB) muscle vs. torque relationship at incremental muscle contraction found non-significant difference [*p* > 0.05 (8)] in their relationship with torque. Compared to EMG, MMG is immune to artefacts from electrical cabling or recording equipment, insusceptible to skin sweating, and sensor-skin impedance mismatch (9), and eliminated the needs for extensive skin surface preparation before electrode attachment (10). Furthermore, MMG signals can be captured using lightweight accelerometers with a low signal-to-noise ratio, which are insensitive to sensor positioning on any area of the skin surface over the muscle (11). Nevertheless, MMG signals from voluntary muscle contractions is prone to interference with crosstalk from adjacent muscles (12), making it difficult to fully isolate specific muscle activities at a joint. To address this gap and obtain muscle-specific behaviors at low intensity of muscle activity, neuromuscular electrical stimulation (NMES) has emerged as a promising approach for isolating specific muscle behaviors (13). Furthermore, NMES-MMG is more sensitive to neuromuscular changes improving the accuracy of torque estimation (14). MMG has been proven crucial for upper limb mobility assessment and supports the development of MMG-based muscle strength models capable of capturing real-time muscle fiber oscillations (15). Unlike Hill-type models, which requires complex mathematical assumptions about muscle tendon units, MMG based torque estimation models eliminates these constraints (16), which are also time-consuming burdening the research (17). However, the inherent magnitude attenuation with the sensor mass, adipose tissue and lower frequency band (5–100 Hz) of MMG signals poses challenges in the direct correlation of muscle efforts and evoked contractions (18). These constraints motivated recent research to develop machine learning algorithms disrupting the non-linearity and non-stationarity characteristics of MMG signals. These simulation algorithms leverage several MMG features inspired by its direct mapping with joint torque production (13).

Building on these advantages, a recent study employed the RMS and zero crossing rate (ZCR) features from MMG signals of the BB and brachioradialis muscles to estimate elbow flexion force using artificial neural networks (ANN) and multiple linear regression achieving a prediction accuracy of 0.883 (19). Another study (20) developed a RFR model to map MMG and elbow joint torque levels, leveraging RMS, mean power frequency (MPF), and sample entropy features, achieving 0.6828 accuracy on an unseen dataset. To further improve the relationship between MMG signals and muscle strength, the study in (21) integrated RMS, mean absolute value, ZCR, MPF, sample entropy, and band energy features. Complementing these efforts, research in (22) expanded the features set by incorporating MMG frequency band energy, wavelet packet energy, and approximate and fuzzy entropy features and achieved improved root mean square error (RMSE). Despite these advancements, further exploration of the acoustic (23) and Hjorth features (24) is essential given the stochastic nature of MMG signals. However, the inclusion of additional features escalates computational complexity, emphasizing the need to assess feature relevance and redundancy (25).

The use of selected features plays a pivotal role to improve the performance of supervised machine learning models by enhancing the learning speed and the generalization capacity (26). These methods are classified into filter methods that rank the features based on the data characteristics; wrapper methods that use machine learning algorithms to select significant features; and embedded techniques that integrates both feature selection and model predictions in a single process (27).

Supervised machine learning models benefit from the high-dimensional complex dataset for better generalization. Specifically, ANN (19), and support vector regression (SVR) (20) have demonstrated remarkable efficacy in mapping non-linear, high-dimensional relationship between muscle activity and joint torque. While the random forest regression (RFR), an ensemble learning technique, has shown prominence in correlating complex features and observations (28), yet it has not been used to explore torque estimation models during NMES-evoked contraction. Moreover, developing effective machine learning models, require efficient hyperparameter tuning (29).

Recent methodologies have increasingly adopted metaheuristic algorithms, which dynamically adjust search agents based on objective functions. Among these, the Equilibrium Optimizer (EO), a physics-inspired algorithm, has outperformed traditional metaheuristic methods (30). Introduced in 2020, EO's uses a fixed population size which leads to candidate solutions getting trapped into the local optima. To address this, in 2021, Jingwei Too introduced the General Learning Equilibrium Optimizer (GLEO), which allows the particle candidate to learn from multiple dimensions to explore promising regions of the search space (31). In 2023, a bi-phasic mutation scheme was evaluated across the k-nearest neighbor algorithm (KNN), support vector machine (SVM), random forest, and discriminant analysis (32). While GLEO has demonstrated considerable promise in biomedical feature selection, it also holds the potential for optimizing RFR model parameters. Nevertheless, the GLEO framework suitable for both feature selection and hyperparameter tuning has not yet been reported. Thus, there remains a room to explore the use of GLEO for joint torque estimation.

Leveraging the promising performance of MMG signals in muscle strength prediction, and the underlying accuracy factors of the RFR model, this study aims to develop a joint torque estimation model from NMES-evoked MMG signals using RFR. Notably, this study is the first to employ GLEO for tuning the number of predictors used at each split of NMES MMG features (*mTrees*), the number of independent variable splits or minleafesize of the tree (*MinLeafSize*), the optimal number of trees (*Ntrees*) and maximum splits of trees (*Nsplits*) in RFR model. Model performance was assessed using the coefficient of determination (R^2^) to capture the correlation between predicted and observed values. Additionally, the RMSE quantified how well the model's predictions align with actual data by assessing the average magnitude of error, and the slope assessed the degree of the model's calibration, revealing the tendency towards overprediction or underprediction, ensuring minimal bias of the model across specific datasets.

The performance of the developed model was benchmarked against state-of-the-art models, including the backpropagation neural network (BPNN) and SVR. While both BPNN (19) and SVR (20) demonstrated a high estimation accuracy in prior studies, these findings primarily relied on data collected from a single hand posture. Although (33) reported a notable torque estimation accuracy, it is crucial to investigate whether NMES-MMG and torque dataset collected from varied forearm postures and elbow joint angles can yield generalizable results for torque estimation (34). However, while this study did not explicitly evaluate the effects of posture and angle on torque estimation, the performance of the generic model suggests that complex NMES MMG from BB muscle is valuable in musculoskeletal studies. This novel approach could facilitate intelligent biomechanical analysis and provide significant clinical utility and research utility.

## 2 Methods

### 2.1 Subjects

Thirty-six male participants (mean age, 22.24 ± 2.94 years; height 172 ± 0.5 cm; and weight, 67.01 ± 7.22 kg) with no prior history of neuromuscular disorder or surgical procedures voluntarily participated in this study. Participants are limited to middle BMI range excluding overweight and malnutrition categories. Each participant signed a written informed consent following a comprehensive briefing of the purpose of the study. Ethical approval [NMRR-20-2613-56796 (IIR)] was granted by the Medical Research Ethics Committee of Malaysia, Ministry of Health adhering to the principles of the Declaration of Helsinki. The experiment took place at the Laboratory of the Faculty of Electronics and Computer Technology and Engineering, Universiti Teknikal Malaysia Melaka (UTeM), Malaysia.

### 2.2 Experimental protocol

Participants attended the experiment on three separate occasions. The first visit involved collecting anthropometric data for the placement of NMES and familiarization with performing maximum voluntary isometric contraction (MVIC) at 90° of elbow flexion. An error margin of 5% or less was allowed between two consecutive MVIC trials, with a minimum of 5 min of recovery between trials to avoid muscle fatigue.

Subsequently, participants familiarized themselves with the sensation of the electrical signal. A motor point at the BB was located using a pen electrode and a protocol of 30 Hz frequency, 110 µs pulse width, and 30 mA current amplitude was determined to be comfortable for all subjects. Participants refrained from vigorous muscle activity for at least 24 h before each subsequent NMES session. Participants unable to reach about 15% MVC of equivalent NMES were excluded from the study (34).

During the experiment, the forearm was positioned in either neutral, pronation, or supination postures of the hand and secured to a customized wooden lever arm. The angle of elbow flexion was adjusted to 10°, 30°, 60°, and 90°, measured using a digital goniometer. Following the guidelines of the International Society of Electromyography and Kinesiology (ISEK), a self-adhesive electrode (4 cm × 4 cm, Hercusense TENS/EMS, V2U Healthcare Pte. Ltd., Singapore) was applied to the motor point, labelled with an indelible pen, while the distal electrode was positioned at the opposite end of the BB muscle belly. Electrical Muscle Stimulator (EMS 7500, V2U Healthcare Pte. Ltd., Singapore) was used to administer stimulation intensity to the BB muscle. The position of the muscle belly was identified through palpation with the elbow flexed at 90°, ensuring accurate placement of the MMG sensor.

**Figure 1:**
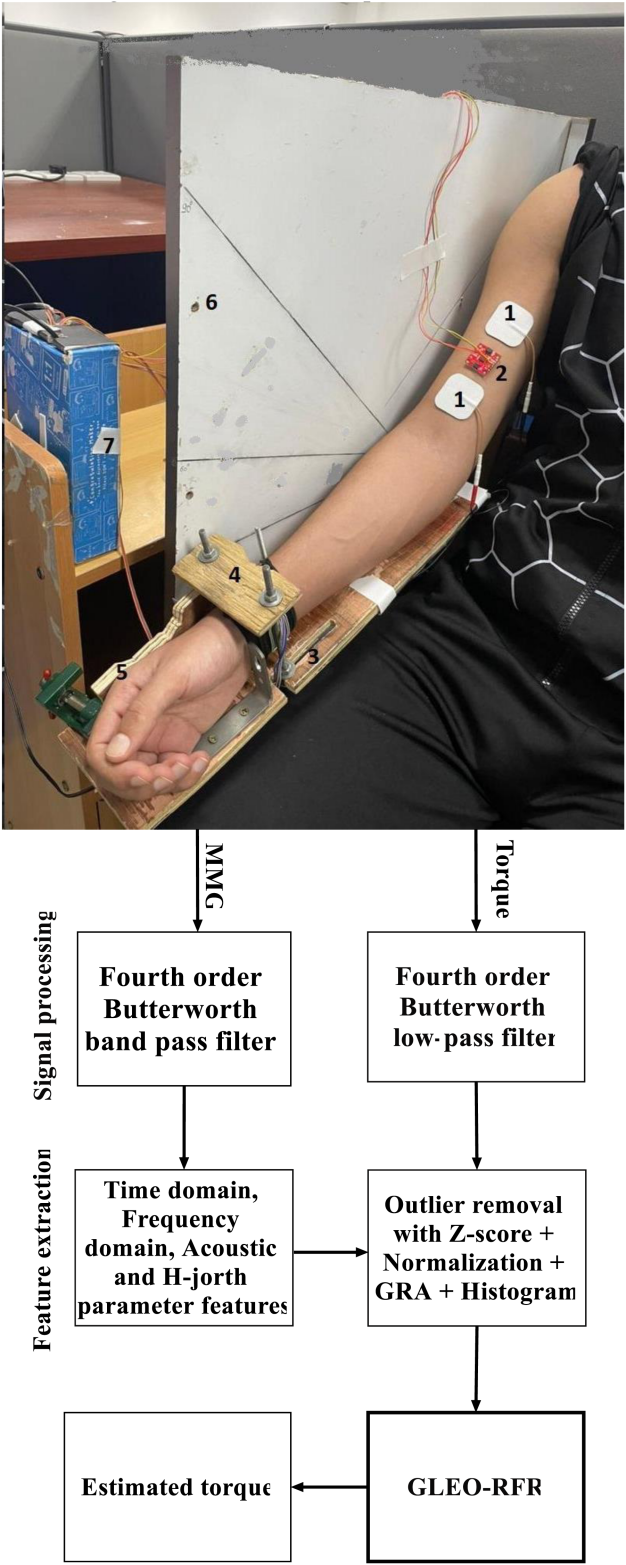
Block diagram of the proposed GLEO-RFR model illustrating key components: (1) electrical stimulation electrode pads, (2) MMG transducer, (3) adjustable lever arm (defined by forearm length), (4) force sensor fixture (underneath brace), (5) wrist posture fixture, (6) elbow joint angle fixture, (7) force and acceleration interface connected to the computer, and the signal processing steps for torque estimation.

For consistency, the lever arm was standardized across all participants using an adjustable table fitted with vises and screws, ensuring the same setup across participants as illustrated in Figure 1. Two trained observers were present throughout the experiment to oversee and maintain the arm posture and elbow joint angle, thus ensuring accurate and ethical data collection. Participants were provided with a 10-minute rest period between posture or angle adjustments, and an additional 5-minute rest between consecutive trials to promote muscle recovery. Each trial lasted 30 s, and participants contributed two recordings per configuration taken at each of four angles (10°, 30°, 60°, and 90°) and 3 forearm postures (neutral, pronation and supination).

### 2.3 Data acquisition and signal processing

Acceleration and Torque signals were concurrently recorded using a customized LabVIEW program (NI LABVIEW 2021, 64-bit) at a sampling rate of 1 kHz. An Arduino Uno R3 was interfaced with acceleration and force transducers. Acceleration data were acquired using a 3-axis digital accelerometer, ADXL-313 model (weight <2.6 g), featuring a flexible and reconfigurable range, operates from 10-bit resolution (±0.5 g) to 13-bit resolution (±4 g) across its three axes. Capable of maintaining full range resolution across any g-range, it effectively captures muscle fiber contraction propagating in three directions at frequencies ranging from above 5 to 100 Hz. The accelerometer was sourced from SparkFun (Colorado, USA).

The ADXL-313 sensor was affixed to the muscle belly using 3MTM VHBTM 4920 double-side adhesive tape (Center St. Paul, MN, USA) (Figure 1). To account for the static acceleration across varied elbow flexion angles and forearm postures, and emphasize muscle activation dynamics, a zero-g bias correction was applied using built-in offset registers on the ADXL-313 sensor. Torque was measured using a force transducer (FS2050 Compression LC1500 GRAM, TE Connectivity, Schaffhausen, Switzerland) and the lever arm length was determined by measuring the distance from the olecranon process to the styloid process of the ulna. The dataset consisted of acceleration signals across three axes and torque information corresponding to different postures and angles and was stored on a hard disk in a personal computer for offline processing.

To suppress the transients' effects associated with torque development and relaxation, the first and last 6 s of each recording were excluded, retaining 18 s for processing as input to the torque estimation model. The power spectrum of MMG signals is well known to be below 100 Hz. MMG data underwent preprocessing by applying a fourth-order Butterworth bandpass filter of 5–100 Hz to the acceleration signals, effectively eliminating artefacts from electrical cabling and body motion. Additionally, torque data were filtered using a fourth-order Butterworth low-pass filter with a cutoff frequency set at 5 Hz (see Figure 3).

### 2.4 Feature extraction

The physiological features in both the time and frequency domain were extracted, alongside acoustic, H-Jorth mobility parameter, and the energy across each frequency band of MMG to explore the relationship between NMES MMG and torque signals. Specifically, the RMS of torque signals, as well as RMS, MPF, median frequency (MDF) and ZCR of MMG signals were computed. The RMS value of MMG reflects the number of active motor units, MPF correlates with the motor unit recruitment and firing rates, MDF indicates the contraction speed with force production (34), and ZCR reflects changes in muscle contraction dynamics. Additionally, features including spectral flatness (SPFlt), spectral spread (SPsp), spectral centroid (SPC) (23), spectral flux (SPFlx) (35) along with Hjorth mobility parameter (24) were also calculated to characterize how the muscle responds to sustained stimulation and evaluate changes in muscle fiber recruitment. Energy contributions from muscle relaxation (5–12 Hz), slow twists motor units (12–40 Hz) and fast twists motor units (40–100 Hz) were also computed (36) to measure the dynamic states of muscle fibers required for optimizing MMG and torque.

To extract detailed information for training RFR, SVR, and BPNN models, a sliding window of 100 ms with a 50% overlap was used for the 12 features from NMES MMG signal length. Each feature of the NMES MMG signal consisted of 311,176 segments. Prior to developing the MMG-Torque models, the uneven magnitude of NMES MMG features, and elbow joint flexion torque were normalized using the *Z*-score method (37). However, the *Z*-normalized datasets exhibited outliers that could affect the prediction. Therefore, the segments with *Z*-score exceeding ± 3 were eliminated (38). The remaining 292,122 segments for each dimension were normalized between 0 and 1 before being used for NMES torque estimation.

**Figure 2:**
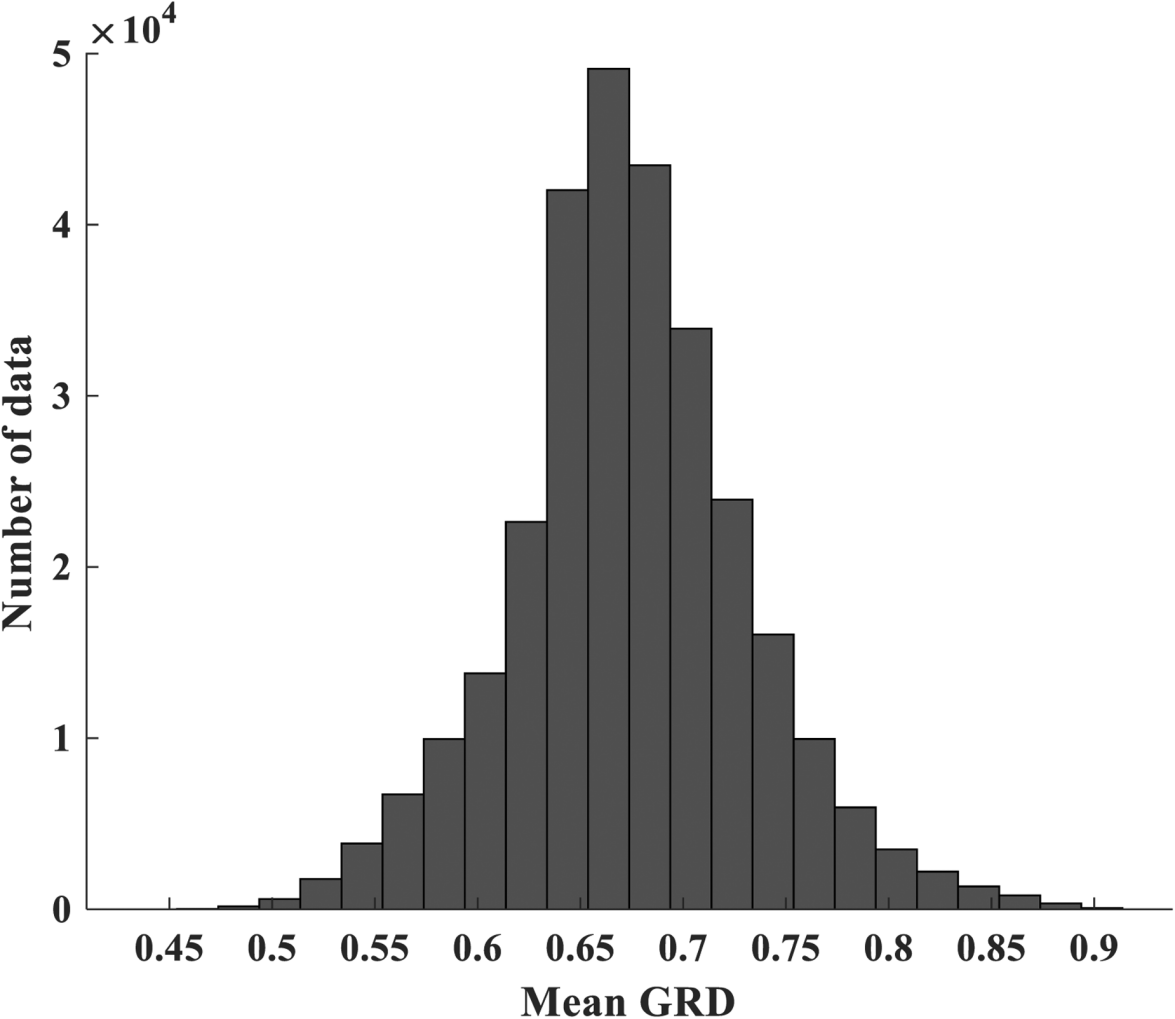
Mean correlation (mean GRD) of MMG and torque data. The amount of data in each bin is obtained from indices of torque and MMG data whose correlation falls into the given minimum and maximum GRD bounding each bin.

An inspection of the grey relational degree (GRD) revealed a moderate to high correlation between each NMES MMG feature and elbow joint flexion torque value (37) as shown in Figure 2. When feeding these features into machine learning models, it is crucial to ensure an equal percentage of data distribution to avoid bias caused by disproportionate feature representation. Data binning was employed, which groups NMES MMG features into bins at similar frequencies of occurrence (39) ensuring even distribution of the features before being processed by the machine learning model.

**Figure 3:**
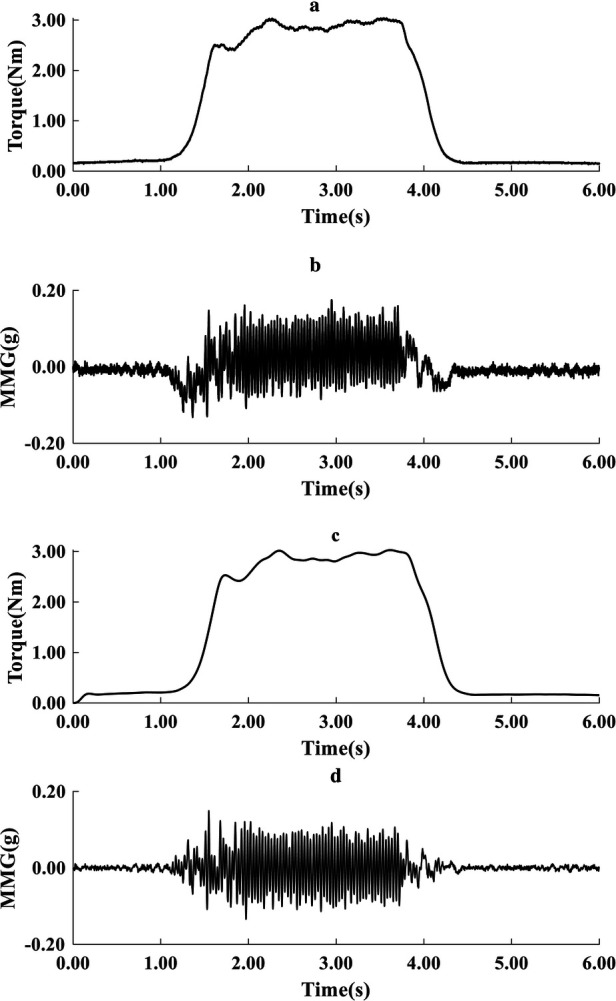
Typical representation of raw torque **(a)** and acceleration MMG signals **(b)**, and filtered torque **(c)** and filtered MMG signals **(d)** during one cycle of recording (6 s).

### 2.5 Random forest regression model

A RFR model leverages an aggregation of decision trees to make predictions. Each decision tree is an hierarchical set of decisions systematically structured from the root to the terminal node of the tree. Each tree in the forest is built using a random subset of input features (40). At each node of the tree, a split point is selected from the training data to maximize information gain. This splitting continues recursively until a minimum node size is reached.

In this study, a subset of data was randomly chosen from 12 input features to build a binary tree structure, where each internal node represents a decision based on a specific feature and threshold, and each leaf node represents the outcome or final label. The ensemble of trees works independently to estimate the output for new data points, enabling the model to efficiently explore the complexity of the data. The final estimation is made by averaging the combined estimations from all trees. The splitting of data at each tree node continues until the lowest RMSE is achieved.

The developed RFR model involves four key parameters namely the *Ntrees* in the forest, *mTrees*, *MinLeafSize*, and *Nsplits*. The default *Ntrees* is 500 (40), with the *mTrees* value set to one-third of total input variables and *MinLeafSize* set to 5. However, the optimization of these parameters was based on the initial values empirically determined in a pretest session, which gave *Ntrees* from 200 to 1,500, *mTrees* of 1 to 12, *MinLeafSize* of 1 to 10, and the *Nsplits* from 100 to 200.

### 2.6 GLEO on MMG feature selection and hyperparameter tuning of RFR (GLEO-RFR-FS-HT)

The bootstrap sample was randomly chosen from the 12-dimension dataset. At each tree node, the most promising split of MMG features was built from the root node based on the lowest RMSE between the observed and predicted torque labels during the training phase. The data-splitting process continued until the final stopping criteria were satisfied.

70% of data was allocated for the training and 30% was used to test the performance of the model on unseen data. This data split was stratified using histogram-based binning to ensure equal percentage of distribution of NMES MMG and torque data (39), as illustrated in Figure 2. We have chosen 50 bins with a bin size of 0.02 whose training resulted in a low RMSE for both the training and testing experiments. This process helped to detect overfitting and provides confidence in the model's estimation performance in real-world scenarios. The MATLAB software (MATLAB® 2022b MathWorks, Inc., Natick, MA, USA) installed on a 64-bit operating system on Windows 11, 12th Gen Intel(R) Core (TM) i7-12700 2.10 GHz, was used for the signal processing and model development.

Optimizing the RFR model involves the feature selection and tuning of its hyperparameters. Due to the vast number of possible combinations of RFR parameters and reliable NMES MMG features, GLEO was used to identify the best features and hyperparameters configurations. For each portion of the training subset, the decision variable RMSE was utilized to select the NMES MMG signal features and the parameters of the RFR (*Ntrees, mTrees, MinLeafSize*, and the *Nsplits*) were recorded. MMG signal feature was selected if its corresponding flag was greater than 0.5. This process was repeated for every random combination of feature subset and RFR hyperparameters, converging on the best fitness value after 100 iterations. The optimal set of NMES MMG features and the RFR hyperparameters were noted and used for the final model performance evaluation on the test subset.

Given the goal of combining feature selection and hyperparameter tuning in RFR model, the GLEO-RFR and EO-RFR models were developed and assessed for their effectiveness in feature selection, hyperparameter tuning, and a combination of both using a specified performance measure (fitness), which is typically the RMSE of the model trained using the NMES MMG features and hyperparameters of RFR at each iteration *t*.

(1) $$RMSE_{i}^{t}=\;\sqrt{\frac{1}{n}}\overset{n}{\underset{i=1}{\sum }}\;(y_{i}-y_{i}^{\prime })$$

where *n* is the number of observations, $y_{i}$ and $y_{i}^{\prime }\;$ are the observed and the predicted target values respectively for observation *i*, $RMSE_{i}^{t}$ measures the deviation between the estimated values $y_{i}^{\prime }\;$. and the observed values $y_{i}$. Lower RMSE values indicate better model performance, with a value of 0 indicating perfect estimation. In this study, fitness is evaluated on the training subset during the model development.

The hybrid feature selection and hyperparameter tuning GLEO-RFR-FS-HT) model begins with initializing the parameters of the GLEO and setting up the candidate population solution consisting of the four hyperparameters of RFR (41) and MMG features flags (31). Next, the algorithm selects the top four candidate solutions based on their performance and calculates their average to form an equilibrium pool. A candidate is then randomly chosen from this pool, and its corresponding index is recorded. Each vector solution is updated iteratively, with the optimal MMG features and RFR tuned hyperparameters being identified and outputted.

**Figure 4:**
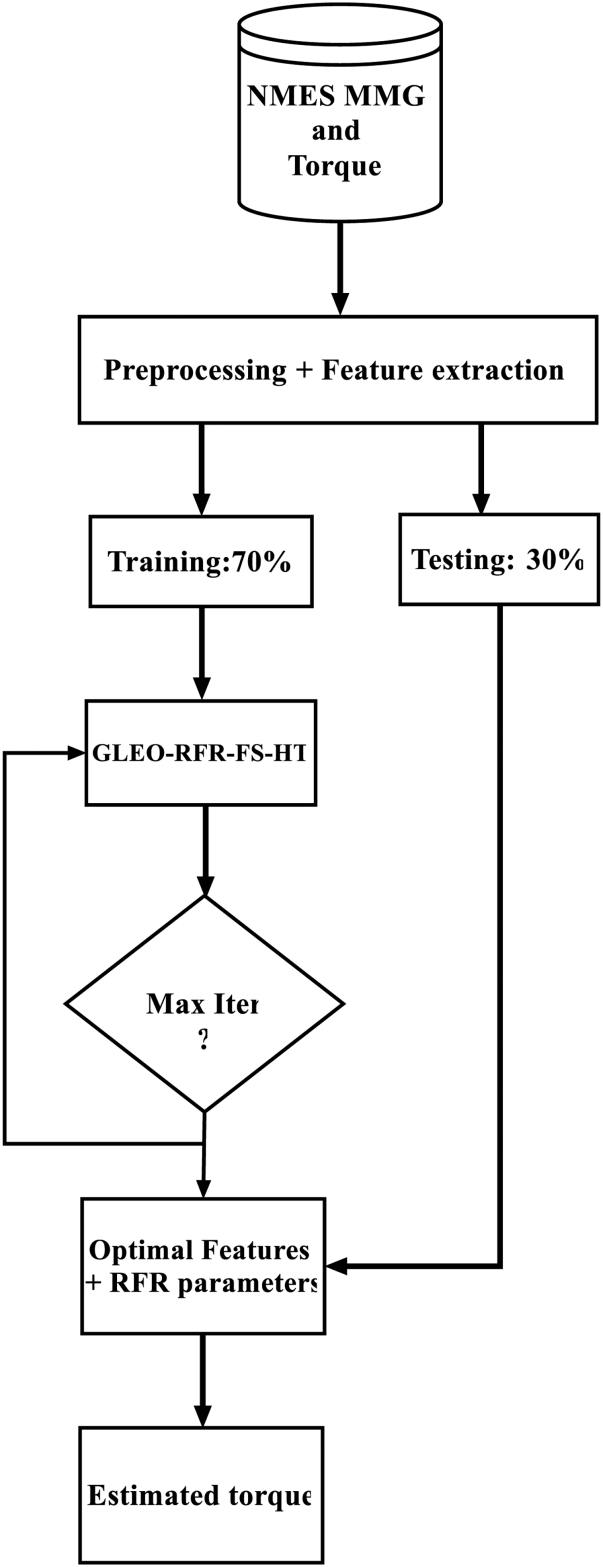
Flowchart of the proposed RFR model for hybrid feature selection and hyperparameter tuning for 100 training iterations (Max iter).

As depicted in Figure 4, the proposed algorithmmences with a preprocessing stage, where NMES MMG and torque data are filtered, relevant NMES MMG features are specified, and outliers are removed. Subsequently, 70% of the dataset is allocated for training the model. Feature selection and hyperparameters tuning for RFR are then conducted at each time the candidate solution trains the RFR model. After 100 iterations (Max Iter), the optimal features and RFR hyperparameters are determined. Thereafter, the tuned RFR model is used to assess the estimation accuracy using unseen test dataset.

The R^2^, the slope and RMSE are used to evaluate the accuracy of estimation, the goodness of fit and the prediction error respectively. Higher R^2^ and slope values along with lower RMSE values indicate improved precision and accuracy in correlating the observed and estimated torque value with the proposed model.

## 3 Results

We devised three distinct models for feature selection, hyperparameter tuning, and a hybrid model integrating both using GLEO. To assess the effectiveness of these GLEO-based approaches, the initial mean values of RMSE, R^2^ and slope values were calculated before feature selection and hyperparameter tuning. For the training subset, these metrics were 0.0963, 0.8882, 0.7903, respectively, while for the testing subset, the corresponding values were 0.1330, 0.7228, 0.6946.

Tables 1–3 present the performance metrics for different approaches of feature selection, and hyperparameter tuning of the RFR model. Notably, the performance metrics of the developed hybrid GLEO for NMES feature selection and hyperparameter tuning improved to RMSE of 0.0461, R^2^ of 0.9665, and slope of 0.8983 respectively for the training subset and RMSE of 0.1174, R^2^ of 0.7853 and slope of 0.7414 for the testing subset. The improvement in R^2^ and the slope was accompanied by a significant decrease in RMSE.

**Table 1:** Perfomance of the RFR model based on GLEO.

Model	R^2^	RMSE	Slope
RFR	0.7228	0.1335	0.6910
GLEO-RFR-FS	0.7265	0.1298	0.6896
GLEO-RFR-HT	0.7573	0.1253	0.7060
GLEO-RFR-FS-HT	0.7853	0.1174	0.7391

HT, hyperparameter tuning; FS, feature selection.

**Table 2:** Performance of the RFR model based on EO.

Model	R^2^	RMSE	Slope
RFR	0.7228	0.1335	0.6910
EO-RFR-FS	0.7255	0.1298	0.6896
EO-RFR-HT	0.7443	0.1282	0.6986
EO-RFR-FS-HT	0.7625	0.1281	0.7021

**Table 3:** Comparison of proposed GLEO with GRD.

Feature	GRD	Ranking by correlation	Ranking by GLEO
RMS	0.8173	1	1
High band	0.7752	2	2
Lower band	0.7608	3	3
Tremor	0.7456	4	NS
SPFlx	0.7261	5	4
MDF	0.6598	6	5
SPC	0.6261	7	6
SPsp	0.6259	8	7
MPF	0.6093	9	8
ZCR	0.5947	10	NS
Mobility	0.5782	11	NS
SPFlt	0.5734	12	NS

NS, not selected.

The model demonstrated improved estimation accuracy, achieving a 33.33% reduction in the feature size. These performance metrics were derived from the optimized RFR model, which comprises *Ntrees* of 847, *MinLeafSize* of 1, *mTrees* of 4, and *Nsplits* of 46,942. The results were compared with those of hyperparameter tuning of RFR for biological dataset (see Table 4), and with the extended version of EO for feature selection as described in the literature (31). The comparisons encompassed various EO extensions including the equilibrium optimizer with a divided population based on distance factor (46), gaussian (47) and biphasic mutation (32).

**Table 4:** Hyperparameter tuning of RFR for biological dataset.

Ref.	Dataset	Method	Observation
(42)	Breast cancer data	Semi-automatic parameter adjustment	Pre-tuning R^2^ of 0.7078 was reported. Post-tuning R^2^ 0.7453 was reported.
(43)	Torque and sEMG data	10-Fold cross-validation and Grid Search	Post-tuning R^2^ of 0.74 ± 0.05, 0.72 ± 0.05, 0.69 ± 0.06, and 0.61 ± 0.06 respectively for four, three, two and one FMG band were reported. Pre- tuning R^2^ were not reported.
(44)	Forces data	PSWO, SSA, GA,	Post-tuning R^2^ of 84.4 was reported. Pre-tuning R^2^ was not reported.
(20)	MMG and torque data	Hilbelt 10-Fold cross-validation	Post-tuning R^2^ of 0.68 was reported. Pre-tuning R^2^ was not reported.
(45)	sEMG and joint angle data	RFR-PCA	Pre-tuning and post-tuning tuning R^2^ values were not reported.
Ours	MMG and torque signal data	Grid Search	Pre-tuning R^2^ of 0.7228 was reported. Post-tuning R^2^ of 0.7327 was reported.
	MMG and torque signal data	GLEO	Pre-tuning R^2^ was 0.7228 and post-tuning R^2^ was 0.7853.

**Figure 5:**
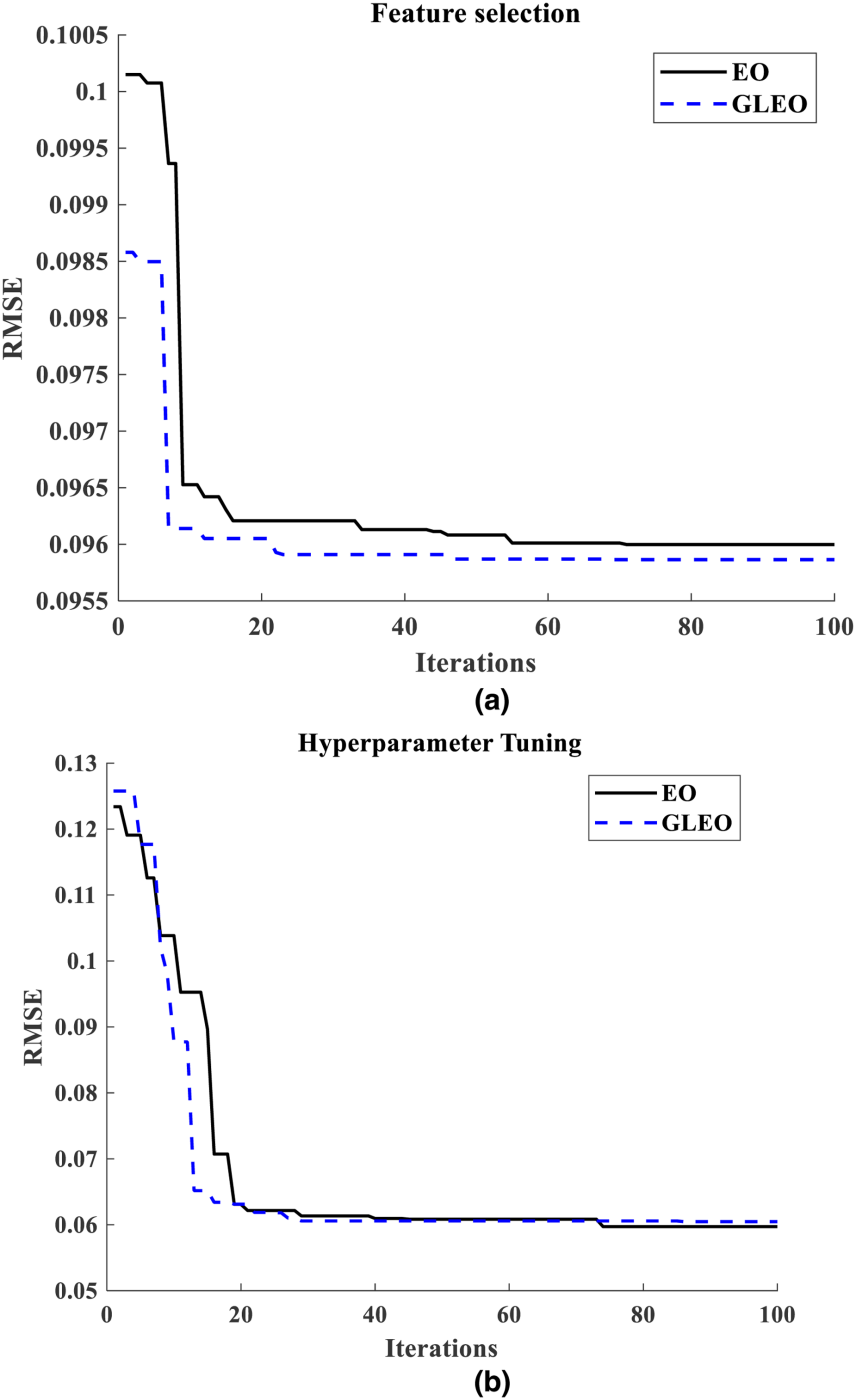
**(a)** training curve of feature selection using EO and GLEO, **(b)** hyperparameter tuning using EO-RFR and GLEO-RFR.

**Figure 6:**
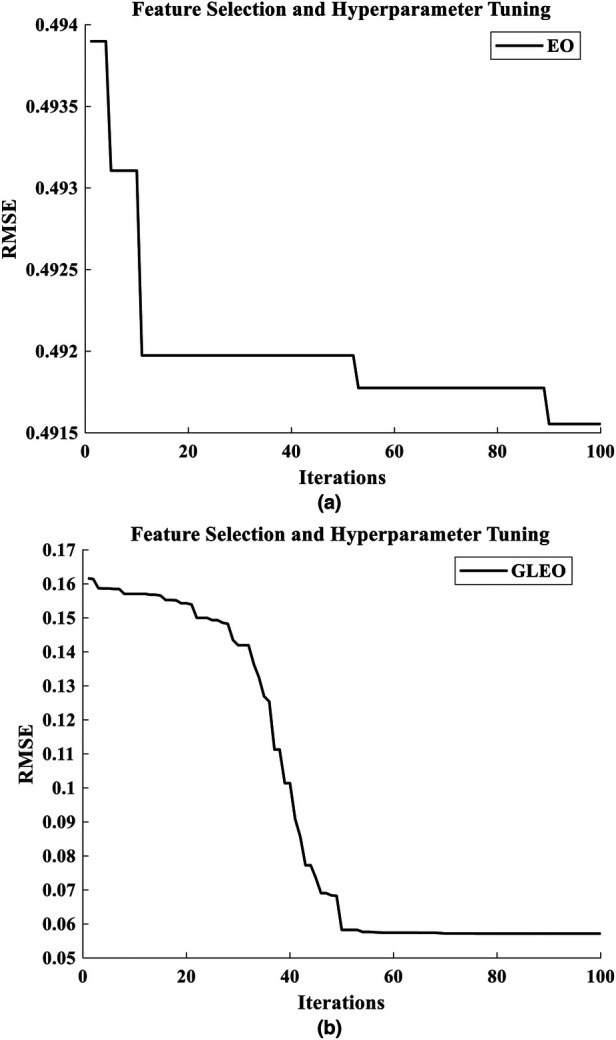
Training curve for hybrid feature selection with hyperparameter tuning using **(a)** EO-RFR and **(b)** GLEO-RFR.

The results from the literature underscore the reliability of GLEO for biological feature selection based on the wrapper method. Specifically, Table 3 highlights the capability of the model to select high informative NMES MMG features which accurately reflect muscle physiology and related biomechanical activity. Figure 5 provides a comparative illustration of the convergence error during training process of EO-RFR and GLEO-RFR for both feature selection and hyperparameter tuning, as well as their hybrid combination. Figures 5 demonstrate that GLEO consistently achieved the lowest RMSE during the training process. Additionally, Figures 6 depict the training process for hybrid feature selection and hyperparameter tuning respectively using EO and GLEO. The training curve showed smooth trend and improved RMSE with increasing training iterations, consistent with the observation in Tables 1, 2.

**Figure 7:**
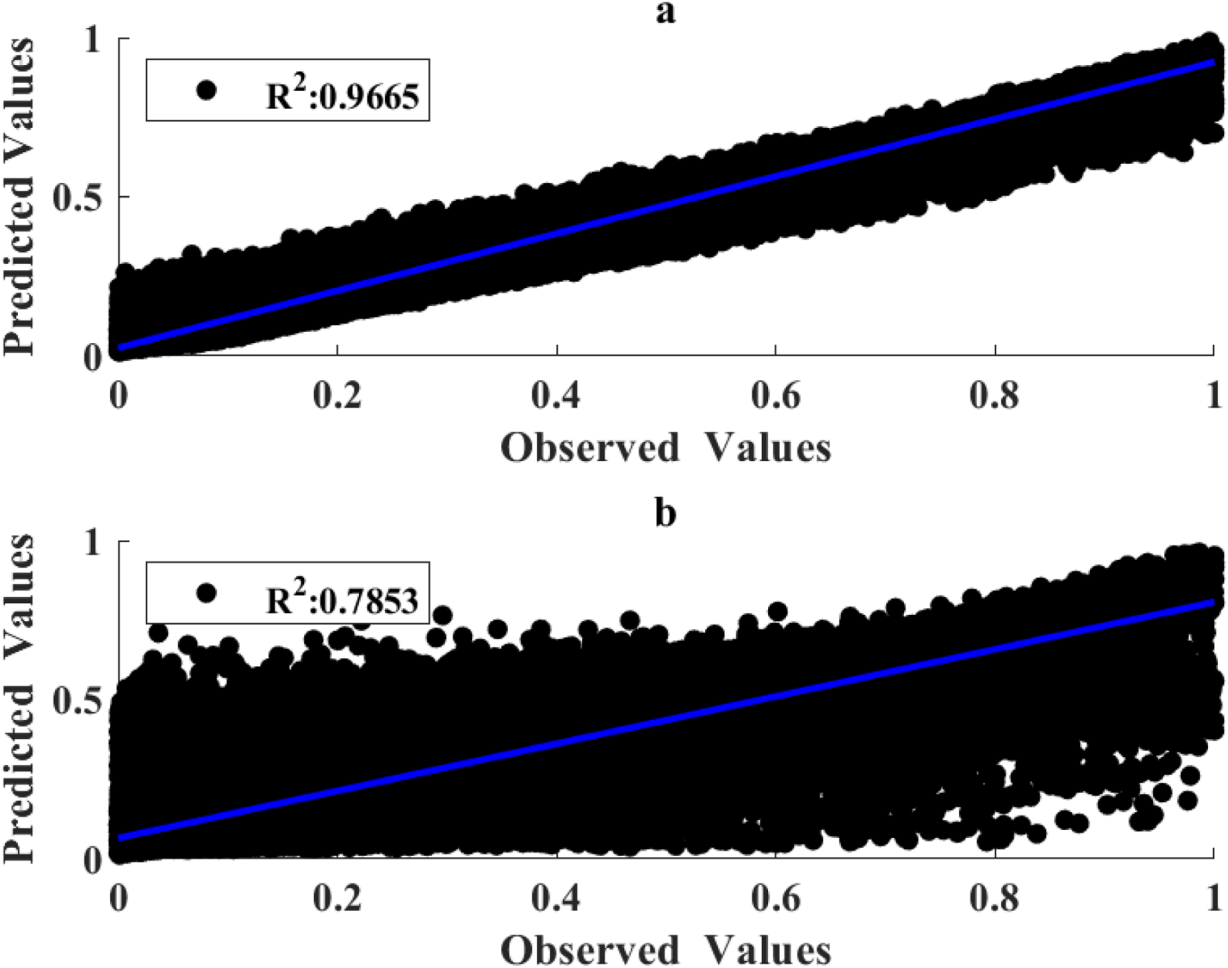
Correlation of measured and predicted torques for the training **(a)** and testing **(b)** subsets using RFR hyperparameters and MMG features obtained by use of GLEO-RFR.

**Figure 8:**
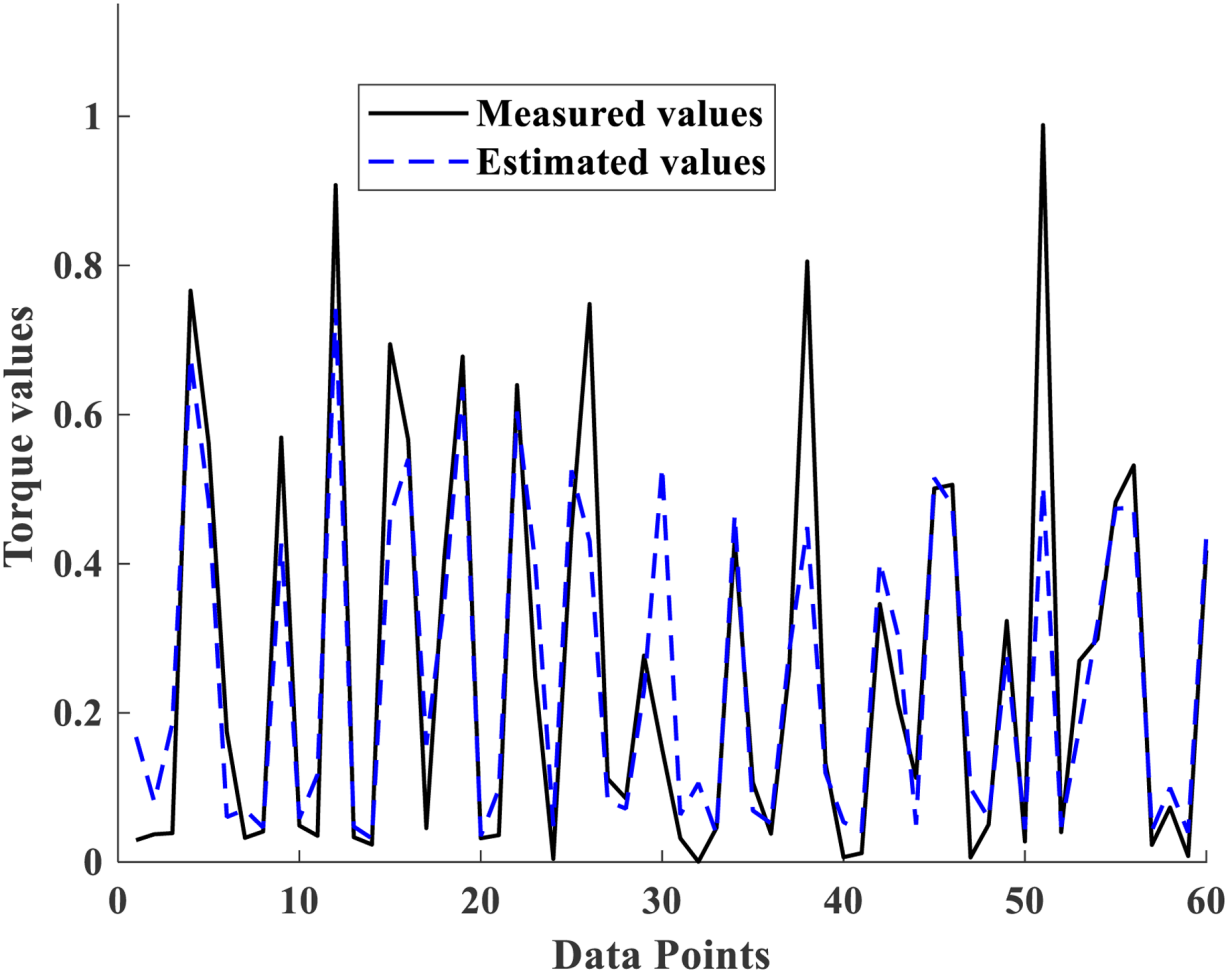
Cross plots of measured torque Vs estimated torque for 60 test subsets.

Taken together, Figures 7, 8 indicate that the results of the proposed GLEO combined with RFR demonstrate its effectiveness in addressing both NMES feature selection and hyperparameter tuning, achieving improved accuracy in complex biological experimental datasets.

### 3.1 Effect of integrating GLEO in RFR models

As seen in Figure 2, the histogram plot indicates that the features of MMG signals exhibited a low to moderate correlation with torque. Tables 1, 2 demonstrate that the GLEO-RFR-FS and EO-RFR-FS yielded improved performance. Furthermore, the GLEO feature ranking from Table 3 reflected the coherence of NMES MMG features and muscle physiology in estimating elbow joint flexion torque. On average, the final model exhibited R^2^ of 0.7853 with a standard deviation of 0.0041.

The model with all 12 features exhibited lower performance metric than the other models with optimal features and RFR hyperparameters. The mean values of the GLEO-RFR-FS-HT model outperformed the other models; with average metrics of 0.7853, 0.1174 and 0.7414 respectively for R^2^, RMSE, and slope.

The scatter and cross plots in Figure 7 illustrate the correlation between measured and estimated elbow flexion torque. This correlation indicates that tuned RFR model leveraging NMES MMG signals effectively estimated the torques from unseen dataset. Based on the performance metrics, the GLEO-RFR-FS-HT model with Ntrees of 847 trees, MinLeafSize of 1, mTrees of 4, and Nsplits of 46,942 emerged as the optimal configuration for achieving a considerable torque estimation.

### 3.2 Comparison of GLEO with other hyperparameters tuning approaches that used biological datasets

In this section, we compared the performance metrics of our model with the other techniques for hyperparameter tuning of RFR-based estimation models found in the literature. Table 4 compares GLEO with state-of-the-art hyperparameter tuning methods regardless of the estimation accuracy but focusing on their improvement. The results highlight that semi-automated mechanisms involving feature selection before hyperparameter tuning are popular. However, these techniques are often time-consuming, prone to error and constrained by the fixed space of model hyperparameters. Interestingly, despite GLEO's novelty in the literature, the results of this research underscore its ability to deliver enhanced outcomes.

In Table 5, we observe the effect of integrating GLEO with the BPNN, SVR and RFR. It is evident that integrating GLEO with RFR provides higher estimation accuracy of 0.7853 compared to 0.5963 for BPNN and 0.5613 for SVR. This shows the efficacy of the RFR model for handling complex biomedical datasets.

**Table 5:** Experimental results from 3 different machine learning models.

Model	R^2^	RMSE	Slope
GLEO-BPNN	0.5963	0.1697	0.5917
GLEO-SVR	0.5613	0.1717	0.5514
GLEO-RFR	**0** **.** **7853**	**0** **.** **1174**	**0** **.** **7414**

BPNN, back propagation neural network; SVR, support vector regression; RFR, random forest regression. Bold indicate the model with the highest performance.

## 4 Discussion

This study presents a GLEO-based framework for MMG feature selection and tuning hyperparameters of the RFR to estimate elbow flexion torque elicited by NMES of the BB muscle in healthy subjects. Distinct models developed using GLEO-RFR (Table 1) and EO-RFR (Table 2) methods were evaluated for their effectiveness in feature selection, hyperparameter tuning and hybrid approach combining both methods. The model's performance was evaluated on unseen data using tuned hyperparameters and selected features by averaging outcomes from 100 testing iterations. All models demonstrated consistent reduction in RMSE alongside improvements in R^2^ and slope values. The hybrid GLEO-RFR-FS-HT model exhibited superior performance evidenced by lower RMSE, higher R^2^ and increasing slope trend, highlighting its capability. These results highlight the model's effectiveness in dynamic and isometric muscle contraction scenarios, where optimal hyperparameters are subject to change in response to the non-stationary nature of the dataset (29).

Previous research has explored torque estimation using MMG features such as RMS, MPF, and ZCR of MMG signals applying artificial neural networks (ANN) model (19). Additionally, SVR model has incorporated the sample entropy and reported favorable performance based on the correlation coefficient and RMSE. A wrapper-based approach using GRD has been employed to identify optimal feature combinations for MMG-based torque estimation (37), achieving a high estimation accuracy. However, the GRD lacks the discriminatory power to discern subtle feature differences crucial for muscle physiology. These models, however, were trained on short segments of stable muscle force and MMG, raising uncertainty about their performance in dynamic scenarios. The GLEO-RFR-FS-HT model addresses the challenging feature selection tasks and achieves improved elbow joint flexion torque estimation accuracy.

Machine learning models rely heavily on hyperparameters, driving the need for various optimization techniques previously deployed for torque estimation using MMG signals features and achieving a high estimation accuracy as measured by R^2^ (37). Conversely, study (48) reported lower R^2^ values of 0.46 for the training and 0.44 for the testing dataset collected from one channel MMG sensor across 6 subjects. The study showed that R^2^ improved with data from multiple channels, though the challenge of accommodating multiple channels on small muscles persists. Furthermore, it is noteworthy that these studies utilized data from a limited number of subjects, potentially affecting the generalization of their findings.

The proposed model, developed using NMES MMG features from a cohort of 36 subjects, achieving an R^2^, slope and RMSE of 0.7853, 0.7414, and 0.1174 respectively on the test dataset. This performance compares favorably to an R^2^ of 0.68 reported for unseen datasets from a previous study (20). The results of the developed algorithm with SVR outperformed the study (48) with R^2^ of 0.5613 and 0.5514 for the training and testing subset. These findings confirm the potential of the proposed approach to handle non-deterministic components in the fitness function of machine learning and dynamic muscle contraction which affect the configuration of optimal hyperparameters at each iteration (29). Despite relying on a high informative channel of MMG sensor, MMG might hold informative features that may have been affected by several factors.

MMG signals provides valuable insights into skeletal muscle activation levels with RMS typically indicating the intensity of motor unit recruitment (49). However, conflicting findings regarding MPF variations with muscle activation levels reflect the complexity introduced by muscle fiber type composition (11, 50), elbow joint angle (34), skinfold thickness (51), muscle size, rate of recruitment and torque development, middle upper arm circumference, upper arm length and body composition (52). While the abovementioned studies have evaluated these factors under varying intensities of voluntary muscle activation, the use of uniform NMES stimulation intensity at the BB muscle in this study obtained varying MPF characteristics at changing elbow joint angle (34). Given the spatial selectivity of MMG signals (53), further investigations are guaranteed to evaluate the performance of the proposed torque estimation model in relation to these factors.

While this study did not quantify crosstalk from other elbow flexor muscles such as the brachialis and brachioradialis, it is likely that NMES has minimized these effects. Given that the brachioradialis is synergetic to the elbow flexion tasks, activation of these muscles across participants and experimental trials could potentially refine torque estimations (54). Moreover, previous research has shown improved performance of estimated torque from multiple sites (48), suggesting that exploration of MMG signals using arrays of transducers could further enhance torque estimation models.

Neurological pathologies such as stroke, spinal cord injury, and neurodegenerative diseases profoundly impair skeletal muscle structure, resulting in muscle atrophy, fibrosis, altered muscle fiber type distribution, and composition in stroke survivors (55). These conditions also compromise the integrity of neuromuscular junction, further exacerbating functional deficits in the upper limbs (56). Given that MMG signals are influenced by the physical characteristics of the muscle being assessed, these pathologies underscore the urgent need for effective intervention strategies to mitigate their defects and promote functional recovery. While the developed model features the ability to process a huge complex data, its implantation into computing systems can process information from arrays of sensors (57) such as inertial measurement unit (IMU). Thus, improves clinical identification of movements disorders and muscle strength estimation required in post-surgical training (58). Advances in assistive technologies have identified muscle re-education using robots as effective way to rebuild lost neuromotor plasticity. NMES promotes neuroplasticity and enhances the expression of various neurotrophic factors, leading to greater axonal growth and the formation of new neuromuscular junctions for atrophic muscle mass in geriatric and loss of physical training of muscles (59). The developed model use myography and force sensors to record the muscle activation, strength, and movement dynamics, and thus estimating the amount of torque needed to support or augment the exoskeleton's movement for walking, lifting, or performing activity of daily living. Therefore, the integration of developed model into the robot-exoskeleton systems may augment the precision and range of motion in disabled limbs (60).

Previous research demonstrated that the spinal excitability to the BB muscle is independently influenced by the joint angle and muscle length, with these effects observed when muscle length or elbow joint angle was maintained constant (61). These observations suggest that MMG data used in this study could similarly be influenced by independent influence of both elbow joint angle and the forearm posture (62). While the existing literature showed that there is no significant difference between joint angle specific and generic torque estimation models (33), this study investigated the capability of GLEO-RFR-FS-HT to select optimal feature from MMG recorded at four elbow joint angle and three forearm postures during elbow flexion tasks. Should the NMES activation of the BB muscle also have received the influence of angle and posture, the performance metrics of GLEO-RFR-FS-HT method needs to be further investigated, given independent limitations identified in this study.

Male subjects were specifically selected due to their typically higher force steadiness and lower variation in motor unit action potential and inter-pulse interval compared to females. Study (63) showed that NMES in young male subjects yield induced %MVIC torque that is clinically significant than female at mean age of 27.6 ± 5.8 yrs. In addition, male tolerated higher phase charge than female for varies NMES for a one-week experimental sessions and improves the tolerance of NMES (64). While the level of NMES induced contraction is muscle dependent, the developed model is useful for the continuous monitoring of the level of muscle susceptibility to NMES intensity using controlled induced % MVC torque and muscle responses detection in young and old male and female individual undergoing controlled NMES training of impaired limb muscles (65).

Taken together, this study focused exclusively on the BB muscle and employed 12 features extracted from the transverse axis of MMG signals. The developed GLEO algorithm extracted 8 features that improved the prediction accuracy. RMS feature reflects the level of motor unit recruitment was used to quantify the amplitude of MMG and force output of the signal which reflect the joint torque (10). Despite a consistent NMES intensity was used, noting the stochastic nature of MMG signals, MPF identified the spectral distribution of the signals, revealing the changes associated with induced slow and fast twitches muscle fiber recruitment. The MDF mirrors changes in muscle recruitment patterns over time course of NMES delivery and divide into two equal halves where a drop in MDF into the lower half can quantify slow twitch muscle fiber recruitment. MPF, and MDF can shift toward lower frequencies during slow-twitch fibers recruitment, but they may not provide the same level of sensitivity to high-frequency components or subtle changes in muscle activation across the entire spectrum. The energy of each MMG band could reflect the dynamics of slow, fast, and resting states of muscle fibers, valuable for optimizing NMES parameters and torque outcomes and monitoring fatigue and recovery. The selected lower and higher energy bands of MMG features confirm the usefulness of NMES to mitigate the muscle tremor, which was identified by the GLEO algorithm. The selected acoustic features include SPC indicative of the global frequency at which the power of MMG signal is concentrated (23), the SPFlx distinguishes the rate of change of MMG signal behaviors over time, and the SPF measures the degree of uniformity in the energy distribution within the frequency domain (66), where abrupt changes in the spectral content reflect the failure to maintain a consistent muscle contraction. The SPsp which measures the energy dispersion across MMG frequencies also reflects the fibers recruitment patterns. Research has shown that Hjorth mobility parameter is sensitive to fatigue state in dynamic contraction (67). MMG in this research was obtained from NMES, which is not varied in temporal dynamics, thus, was not selected for this study as the tremor effect also avoided in the design of the experimental protocol. The introduction of selected acoustic features to the RMS, MPF, and MDF highlights the relevance of these features in torque estimation models. The use of hybrid GLEO is essential and showed the capacity to tune and select significant physiological relevant features. MMG being at infant development stage, these features are useful for future exploration of MMG behaviors on other skeletal muscles.

Evidence suggesting that MMG signals from multiple sites and muscles improve torque estimation performance suggests that future research should incorporate MMG data from additional elbow flexor muscles. Furthermore, the MMG signals in this study were collected from participants with relative, but not identical, anthropometric characteristics, which may imply differences in muscle capacity, fiber type composition, and rate coding. Although equal proportions of data from various postures and angle-specific configurations were used, afferent pathways and individual variations in muscle responsiveness to varied NMES could influence the outcomes. Future studies should address these factors to further evaluate and enhance prediction performance of torque estimation models.

Recent research on assistive technology predominantly relies on modes developed from simulated data, which is unlikely to not reflect underlying muscle physiology. These models often use inverse dynamics, predefined physical laws, and mathematical simplification (68). While polynomial regression can fit the data too well, it lacks the ability to lean relevant features, and requires manipulation of polynomial terms (69). This process is time consuming and prone to numerical instability with increased polynomial degree (70), and struggles with intricate patterns involving multiple interacting variables, where models like random forest excels (45). Despite the prevalence of machine for learning hyperparameter tuning and feature selection (31), a hybrid combination of both was not previously developed using complex physiological and non-physiological datasets. This study proposes GLEO based hybrid approach for feature selection and hyperparameter tuning of the random forest regression and showed improved torque estimation metrics, with the ability to identify underlying physiological features from a complex dataset. The outcomes show the ability to process big data size obtained from wearable sensors for posture recognition (58), post-surgical monitoring, and treadmill muscle re-education (71).

## 5 Conclusion

This study proposes a novel hybrid approach GLEO-RFR-FS-HT for optimizing the hyperparameters of the RFR and identifying optimal subsets of MMG features for elbow flexion torque estimation. The method was tested against various models including SVR, BPNN and RF using NMES MMG signals and torque datasets. The study revealed the capability of the model for selecting optimal MMG signals features and tuned hyperparameters of the RFR yielded significant estimation accuracy, measured by R^2^, slope and RMSE of 0.7853, 0.7414, and 0.1174 respectively on the test dataset. These metrics reveal that the developed model captures the underlying relationship between MMG variables and torque measurements. While the study's findings are promising, it is crucial to acknowledge that the data was from healthy subjects which may influence the model's generalizability. MMG signals from pathological muscles may exhibit differences in amplitude and frequency. Furthermore, the study used data obtained from a single elbow flexor muscle, assessing four angles and three forearm postures. Future research should explore the model's application to MMG signals from pathological muscles, healthy muscles at varying angles and postures, and synergistic muscles involved in elbow flexion, such as the brachialis and brachioradialis. This would enhance the model's robustness and applicability across a broader range of clinical and functional scenarios.

## Data Availability

The original contributions presented in the study are included in the article further inquiries can be directed to the corresponding author because we have no supplementary materials.
